# Protein import into bacterial endosymbionts and evolving organelles

**DOI:** 10.1111/febs.17356

**Published:** 2024-12-10

**Authors:** Megan E. S. Sørensen, Mygg L. Stiller, Lena Kröninger, Eva C. M. Nowack

**Affiliations:** ^1^ Department of Biology, Institute of Microbial Cell Biology Heinrich Heine University Düsseldorf Germany

**Keywords:** endosymbiosis, envelope membranes, mealybug, nitroplast, organellogenesis, *Paulinella*, protein translocation, Strigomonadinae, targeting signals, UCYN‐A

## Abstract

Bacterial endosymbionts are common throughout the eukaryotic tree of life and provide a range of essential functions. The intricate integration of bacterial endosymbionts into a host led to the formation of the energy‐converting organelles, mitochondria and plastids, that have shaped eukaryotic evolution. Protein import from the host has been regarded as one of the distinguishing features of organelles as compared to endosymbionts. In recent years, research has delved deeper into a diverse range of endosymbioses and discovered evidence for ‘exceptional’ instances of protein import outside of the canonical organelles. Here we review the current evidence for protein import into bacterial endosymbionts. We cover both ‘recently evolved’ organelles, where there is evidence for hundreds of imported proteins, and endosymbiotic systems where currently only single protein import candidates are described. We discuss the challenges of establishing protein import machineries and the diversity of mechanisms that have independently evolved to solve them. Understanding these systems and the different independent mechanisms, they have evolved is critical to elucidate how cellular integration arises and deepens at the endosymbiont to organelle interface. We finish by suggesting approaches that could be used in the future to address the open questions. Overall, we believe that the evidence now suggests that protein import into bacterial endosymbionts is more common than generally realized, and thus that there is an increasing number of partnerships that blur the distinction between endosymbiont and organelle.

## Graphical abstract

The acquisition of bacterial endosymbionts by ancient host cells followed by massive adaptation of the partners gave rise to mitochondria and plastids. Today, various systems with younger and less integrated endosymbionts provide insights into the initial steps in the endosymbiont to organelle transition. In this review, we summarize the current evidence for protein import into diverse bacterial endosymbionts which represents a hallmark in this transition process.

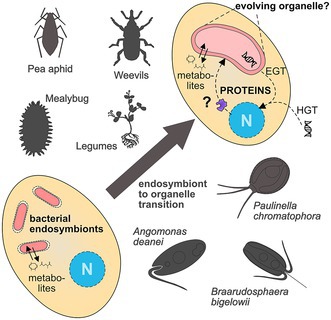

**Abbreviations**
Aa: amino acid(s)
BAM: β‐barrel assembly machinery
BCR: bacteriocyte‐specific cysteine‐rich peptide
crTP: chromatophore transit peptide
Cryo‐EM: cryogenic electron microscopy
DNA: desoxyribonucleic acid
EGT: endosymbiotic gene transfer
ER: endoplasmic reticulum
ETP: endosymbiont‐targeted protein
HGT: horizontal gene transfer
IRLC: inverted repeat‐lacking clade
lCTP: long chromatophore‐targeted protein
LECA: last eukaryotic common ancestor
LPS: lipopolysaccharide(s)
mRNA: messenger ribonucleic acid
MS: mass spectrometry
MYA: million years ago
NCR: nodule‐specific cysteine‐rich peptide
Omp: outer membrane protein
PG: peptidoglycan
RNAi: RNA interference
sCTP: short chromatophore‐targeted protein
SP: signal peptide
SPE: *Sitophilus* primary endosymbiont
Tat: twin‐arginine translocation
TEM: transmission electron microscopy
TIC: translocon of the inner chloroplast membrane
TIM: translocon of the inner mitochondrial membrane
TM: transmembrane
TOC: translocon of the outer chloroplast membrane
TOM: translocon of the outer mitochondrial membrane
UCYN‐A: unicellular cyanobacteria nitrogen‐fixing group A
uTP: UCYN‐A transit peptide
VDAC: voltage‐dependent anionic channel

## Introduction

The acquisition of bacterial endosymbionts more than 1.5 billion years ago and their integration into the intracellular networks of a unicellular host resulted in the evolution of mitochondria and primary plastids (referred to as plastids hereafter). The resulting genetically chimeric organisms (i.e., heterotrophic and photosynthetic eukaryotes) form today a major part of Earth's rich biodiversity. A critical process in organellogenesis, that is, regarded by most as marking the boundary between an endosymbiont and an organelle, is the evolution of protein import systems that translocate nucleus‐encoded proteins across the organellar envelope membranes. This critical step enabled the transfer of hundreds of endosymbiont genes, whose products are essential for metabolism, growth, genetic information processing, and division, to the host nuclear genome in a process termed endosymbiotic gene transfer (EGT) [1, 2]. Today, mitochondrial and plastid genomes encode no more than 66 and 240 proteins, respectively [3, 4].

The majority of nucleus‐encoded proteins, that are imported into mitochondria and plastids, are translated on eukaryotic ribosomes as precursor proteins carrying N‐terminal signal sequences, called transit peptides. The transit peptides interact with soluble sorting factors and chaperones before binding to receptors in the TOM and TOC complexes (short for translocon of the outer mitochondrial and chloroplast membranes) that ‘forward’ the recognized proteins to transmembrane channels within these multi‐protein complexes. The TOC complex forms a super‐complex with the TIC complex (translocon of the inner chloroplast membrane), which translocates the protein across the inner membrane [5, 6, 7, 8, 9]. Proteins imported through the TOM complex can be routed to the TIM23 or TIM22 complex (translocons of the inner mitochondrial membrane). The former delivers the precursor proteins to the inner membrane or matrix, and the latter inserts hydrophobic membrane proteins, particularly mitochondrial carrier proteins with cryptic internal targeting signals into the inner membrane. Other specialized pathways exist for the insertion of outer membrane proteins and import of inter membrane space proteins that largely lack transit peptides. Several recent reviews discuss in detail the current knowledge regarding protein import into mitochondria and plastids [10, 11, 12, 13, 14].

The complexity of the molecular machineries that target, translocate, and sort nucleus‐encoded proteins into mitochondria and plastids, and sub‐compartments therein, likely represents a main hurdle for organellogenesis events and raises the questions of (a) how protein import into organelles started, (b) whether more basic protein import mechanisms exist in more recently acquired bacterial endosymbionts, and (c) would novel organelles that evolve independently use similar mechanisms for protein import as mitochondria and plastids.

A place to search for answers to these questions are the countless eukaryotes from across all superphyla that have acquired diverse, more recent intracellular bacterial symbionts. These endosymbionts equip their hosts with a range of novel physiological functions, including photosynthesis, N_2_ fixation, biosynthesis of essential metabolites or cofactors, chemolithoautotrophy, and anaerobic respiration [15, 16, 17, 18, 19]. In contrast to organelles, these endosymbionts generally have been regarded as ‘genetically autonomous’, that is, they import metabolites but not proteins from their host cells [20]. However, over the last 15 years, patchy evidence started to accumulate that at least some of these ‘endosymbionts’ might not be as genetically autonomous as originally thought. For two cases, specifically the photosynthetic chromatophore of the cercozoan amoeba *Paulinella chromatophora* and, as of this year, the N_2_‐fixing ‘nitroplast’ (or UCYN‐A endosymbiont, short for ‘unicellular cyanobacteria nitrogen‐fixing group A’) in the haptophyte alga *Braarudosphaera bigelowii*, the import of hundreds of nucleus‐encoded proteins has been revealed by protein mass spectrometry (MS) [21, 22, 23]. Imported proteins compensate for the loss of corresponding genes from the endosymbiont genome, characterizing chromatophores and UCYN‐A as novel genetically integrated organelles. When speaking of ‘genetic integration’, we refer to endosymbionts (now called organelles) in which essential endosymbiont genes have been functionally replaced by nuclear genes with products that are imported into the endosymbiont. In contrast, ‘metabolically integrated’ endosymbionts receive only metabolites from the host. However, in addition they may be targeted by host effector proteins that manipulate their behavior in the symbiotic system. The latter endosymbionts should, in theory, be viable outside the host when supplemented with the right metabolites.

In this review, we summarize the evidence for protein import into bacterial endosymbionts. We describe what is known about the cellular integration, the molecular mechanisms underlying protein import (very little), the origin and functions of imported proteins, and reflect on the hurdles for the evolution of protein import into a bacterial endosymbiont.

## Cellular integration of bacterial endosymbionts

Bacterial endosymbionts show diverse modes of cellular integration and subcellular localizations within eukaryotic cells. In multicellular organisms, endosymbionts are typically restricted to specific tissues or organs composed of specialized endosymbiont‐harboring cells, called ‘bacteriocytes’ [15, 18, 24], whereas in single‐celled protists, every host cell contains the endosymbiont(s). Most bacterial endosymbionts are located in the cytosol of their host cell, either free (i.e., surrounded only by its own bacterial envelope) or housed in a specialized host‐derived vacuole. Symbiont and vacuole together are called the ‘symbiosome’. However, some endosymbionts reside in more unusual locations such as the ER [25], the nucleus [26], or the mitochondrion [27]. An extreme case is found in the mealybug, a plant sap‐feeding insect, that contains two matryoshka‐like nested bacterial endosymbionts in its bacteriocytes [28].

**Fig. 1:**
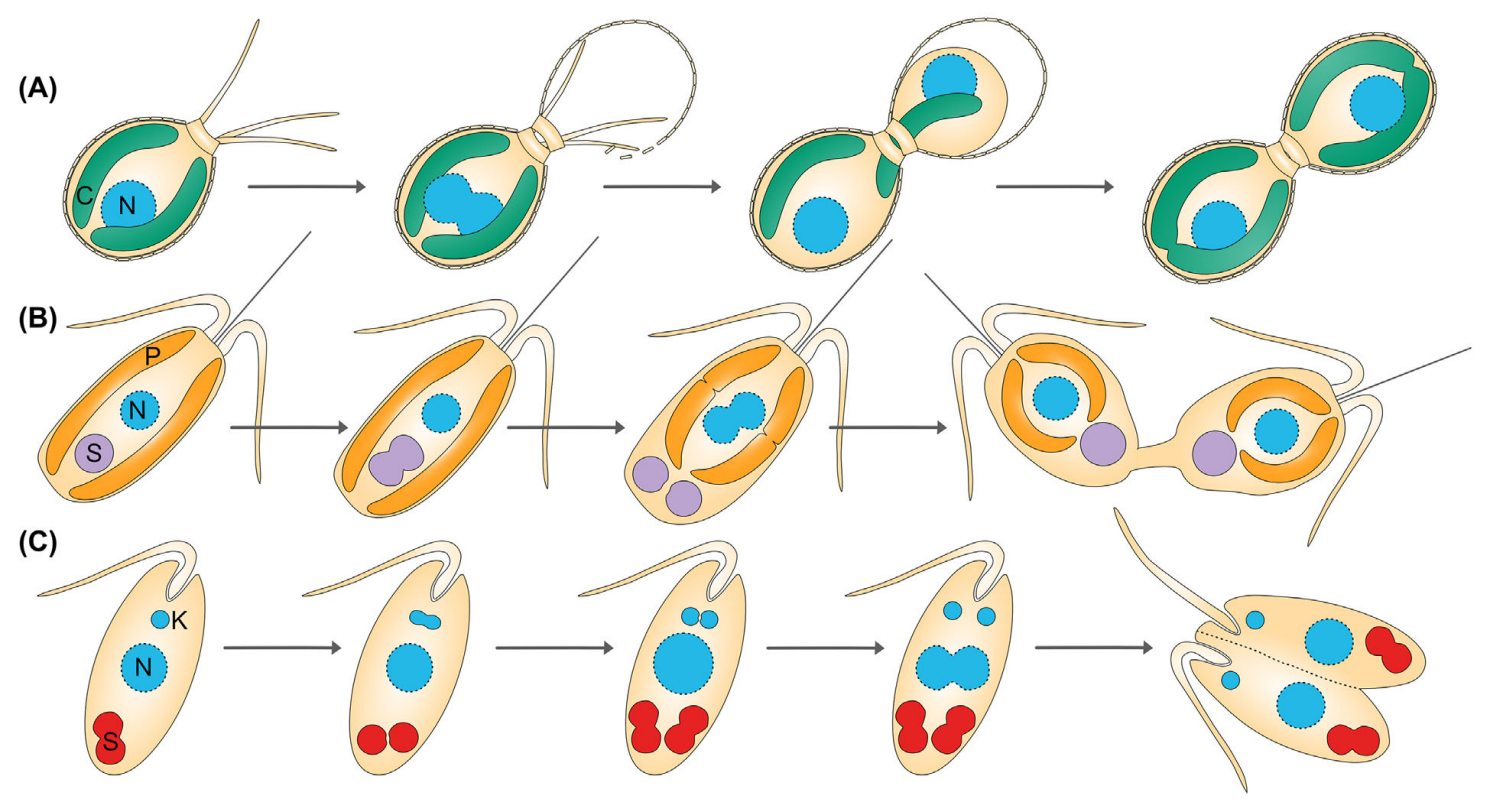
Synchronized cell division of endosymbiont‐harboring protists with their endosymbionts. (A) *Paulinella chromatophora*, (B) *Braarudosphaera bigelowii*, (C) *Angomonas deanei*. C, chromatophore; K, kinetoplast (a network of circular DNA containing many copies of the mitochondrial genome typical for the kinetoplastida); N, nucleus; P, plastid; S, endosymbiont.

Many bacterial endosymbionts are vertically transmitted from one host generation to the next and have coevolved with their hosts for sometimes hundreds of millions of years [29, 30, 31, 32, 33, 34]. In some protists, vertical transmission involves strict synchronization of host and endosymbiont cell division, which establishes a defined number of endosymbiotic units per host cell (e.g., in *P. chromatophora* [35], *B. bigelowii* [23, 36], and in the trypanosomatid *Angomonas deanei* [37]) (Fig. 1). As a consequence of their perpetual confinement to a stable, metabolite‐rich environment, drift, and the long‐time scales of these interactions, the genomes of vertically transmitted endosymbionts tend to reduce, in extreme cases down to 200 or fewer protein‐coding genes [38, 39, 40]. Host complementation of essential symbiont functions relaxes the selection pressure on the corresponding genes, which can eventually lead to their loss, while host dependence upon its increasingly degenerating and reducing endosymbiont can drive it down the ‘evolutionary rabbit hole’ [41]. The complemented products mostly originate from the host itself including re‐assigned organellar support genes, but sometimes originate from EGT from the endosymbiont itself [42, 43, 44, 45, 46] or, more often, from horizontal gene transfers (HGTs) from bacteria other than the endosymbiont [39, 42, 43, 45, 46, 47, 48]. Together, these processes result in highly complementary sets of proteins encoded in nucleus and endosymbiont that, in concert, mediate diverse biological processes.

The molecular mechanisms supporting this tight host/symbiont cooperation are poorly understood. In many endosymbionts, gene losses not only concern metabolic enzymes but extend to proteins involved in genetic information processing (such as DNA replication and repair, transcription, and translation) [48, 49]. Some of these functions that localize in the bacterial cytoplasm appear to be compensated by nucleus‐encoded proteins that are specifically upregulated in the bacteriocytes housing the endosymbionts [48]. Furthermore, although complementary sets of metabolic enzymes in host and endosymbiont suggest extensive, bidirectional metabolite exchange between the symbiotic partners, reductive endosymbiont genome evolution is typically accompanied by a pronounced loss of solute transporters [19, 22, 50, 51, 52, 53, 54, 55]. Both observations suggest that host‐encoded proteins are imported into the endosymbionts and inserted into their membranes. However, whether protein import in deed plays a role for the complementation of lost bacterial functions has not been studied in most systems.

## Restructuring of the bacterial envelope during organellogenesis

Bacteria depend on diverse protein transport systems to release proteins into the periplasm, insert them into the outer membrane (in Gram‐negative bacteria) or transport them out of the cell. The most central are the general translocases of the cytoplasmic membrane: the Sec translocon that secretes unfolded proteins across or inserts transmembrane (TM) proteins into the membrane, and the twin‐arginine translocation (Tat) machinery that transports folded proteins [56]. The β‐barrel assembly machinery (BAM) inserts β‐barrel proteins into the outer membrane [57]. These vectorial transport systems, however, cannot be simply operated in reverse, and bacteria do not generally contain protein import systems that would readily allow for the import of host‐derived proteins. Hence, organellogenesis events must have entailed a major restructuring of the bacterial envelope and protein complexes therein, in order to establish metabolic connectivity and the capacity to import nucleus‐encoded proteins.

If mitochondria and plastids were originally enclosed in a host‐derived membrane, they lost this third membrane [58]. Like the outer membranes of their bacterial ancestors, the outer mitochondrial and plastid membranes contain β‐barrel proteins, but the lipopolysaccharides (LPS) that formed the outer leaflet of the bacterial outer membrane have been lost. Additionally, the peptidoglycan (PG) layer has been lost in mitochondria and most plastids and is only retained in the plastids of Glaucophyte algae, mosses, and some spermatophytes [59, 60, 61].

The central protein‐conducting channels of the TOM complex (Tom40) and the TOC complex (Toc75 and Toc159) are formed by β‐barrel proteins, however, not all of these can be linked to ancestral bacterial proteins. Tom40 shares a common origin with the mitochondrial porin VDAC (voltage‐dependent anionic channel) [62, 63]. The VDAC superfamily apparently emerged only in the context of mitochondrial evolution in the last eukaryotic common ancestor (LECA) by the amplification of a double ββ‐hairpin element; a bacterial precursor protein could not be pinpointed [64, 65]. Toc75 belongs to the Omp85 protein family that also contains BamA, the central protein transporting protein of the bacterial BAM complex [66]. However, Toc75 was recently shown to form a hybrid β‐barrel together with a member of the Toc159 protein family which is believed to have originated with the eukaryotic cell [8, 9, 67]. A phylogenetic relationship between bacterial branched‐chain amino acid transporters, the mitochondrial Tim17, Tim22, and Tim23 proteins (that form the central protein‐conducting channels of the TIM23 and TIM22 complexes), and Tic20 (that likely forms a central part of the protein translocation channel of the TIC complex [10]) that has been initially suggested [68, 69], could not be supported by later studies [70, 71, 72]. Instead, Tim17, Tim22, and Tim23 apparently resulted from gene duplications that occurred before the appearance of the LECA, but their ancestral gene remains obscure [72, 73]. For Tic20, cyanobacterial homologs of unknown function can be identified [74]. Following plastid acquisition, the Tic20 family diversified into two protein groups of which only the faster evolving group 1 could be functionally linked to protein import [71]. In addition to the main protein conduction channels, the TIC/TOC and TIM/TOM complexes contain numerous auxiliary subunits of bacterial and eukaryotic origin [67, 75, 76, 77]. Hence, although the mitochondrial and plastid protein translocation machineries contain a number of proteins of bacterial origin, these proteins underwent extensive changes over time, some probably beyond recognition, and became part of novel multi‐protein complexes of mixed genetic origin that provide functions that do not exist in bacteria.

## The endosymbiont envelope in more recently established endosymbioses

**Fig. 2:**
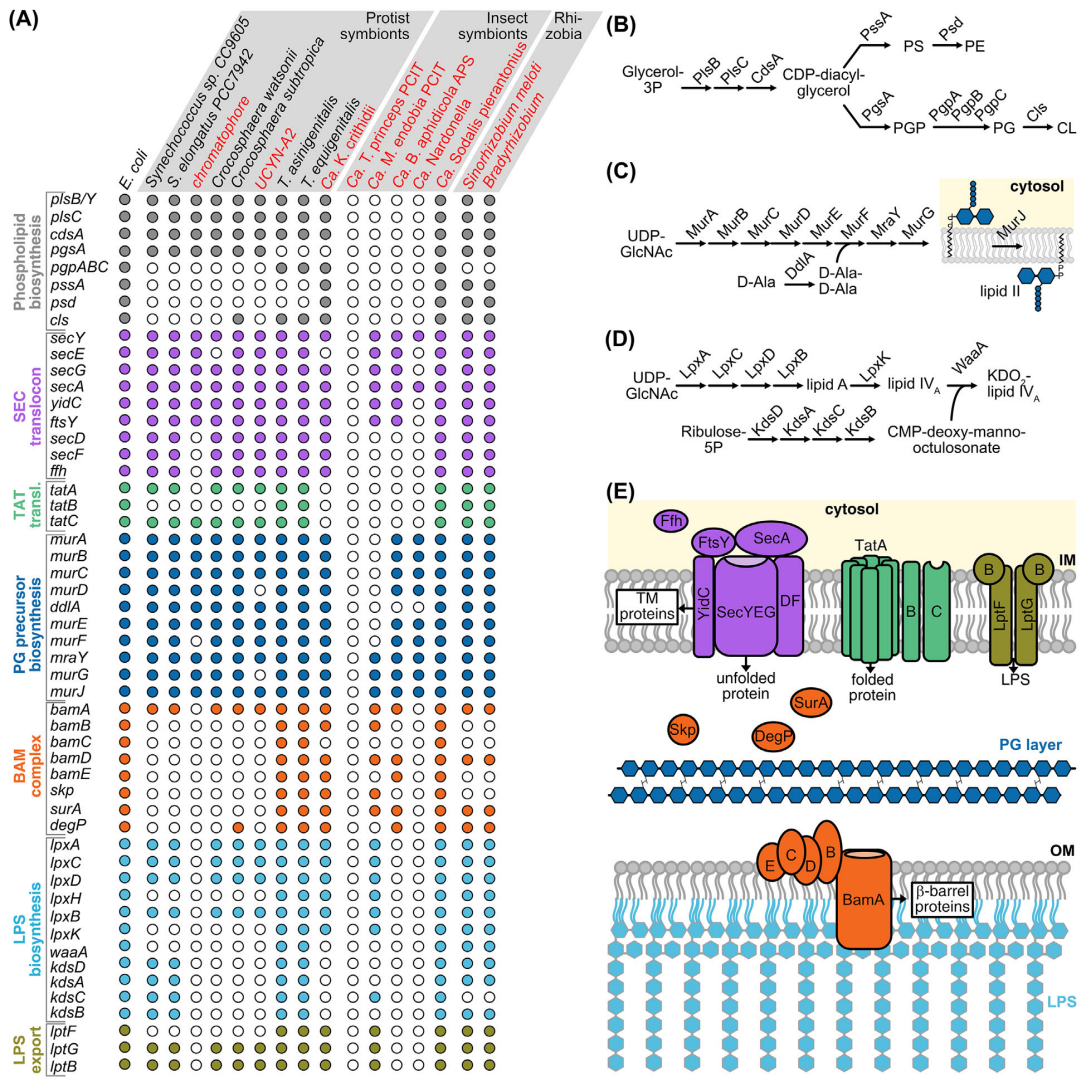
Capacity of endosymbionts to autonomously build a bacterial cell envelope. (A) Gene presence/absence patterns in the genomes of endosymbiotic (red) and free‐living bacteria (black) as analyzed by the toolset provided by the Kyoto Encyclopedia for Genes and Genomes (https://www.kegg.jp/). (B–E) As a reference, pathways for phospholipid biosynthesis (B), PG biosynthesis (C), LPS biosynthesis (D), and the envelope structure of *Escherichia coli* (E) are provided.

**Fig. 3:**
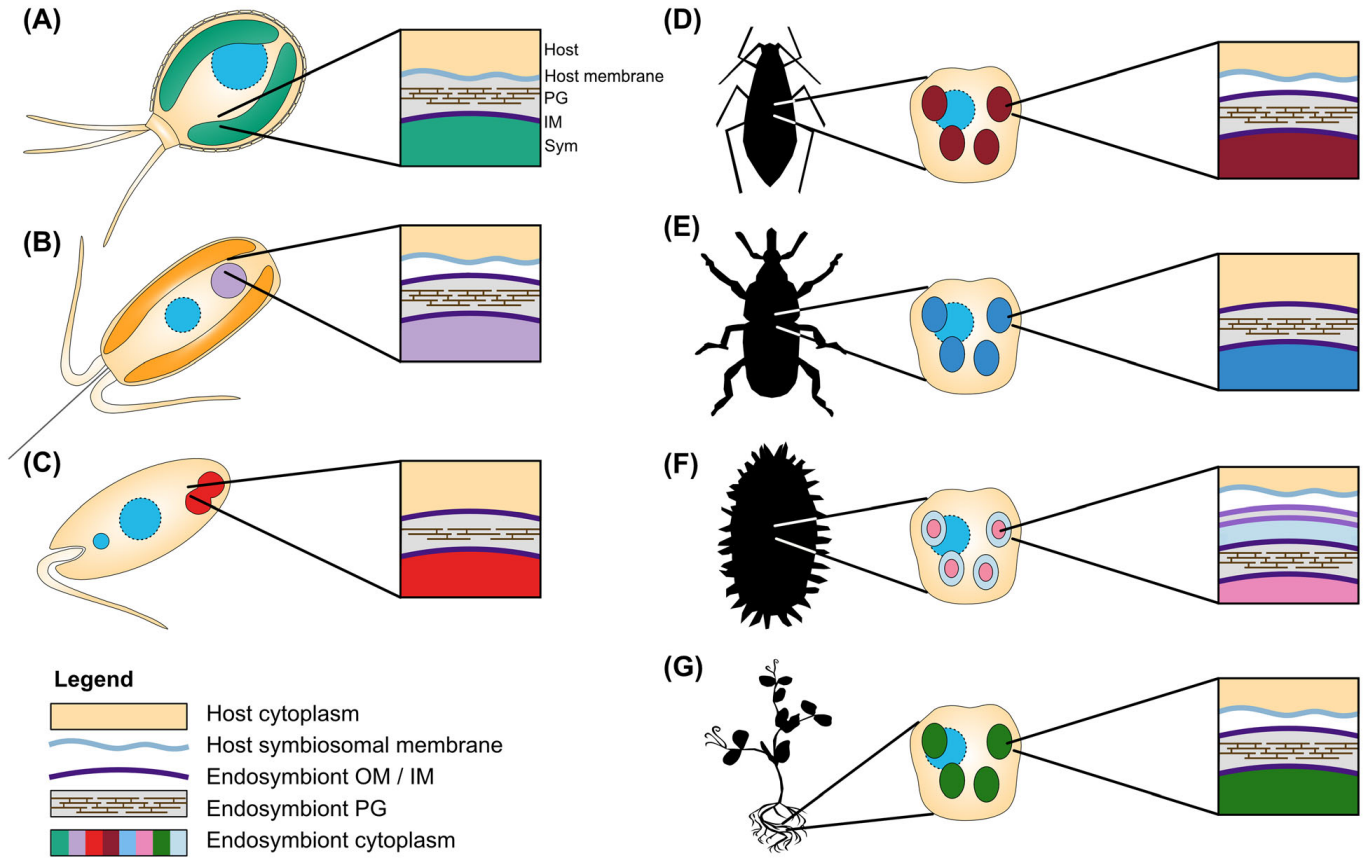
Visualizations of the endosymbiotic systems, highlighting the membrane systems surrounding the endosymbionts that any imported proteins need to traverse. (A) *Paulinella chromatophora* and its chromatophores. (B) *Braarudosphaera bigelowii* and UCYN‐A, nitroplast. (C) *Angomonas deanei*, as a representative of the Strigomonadinae, and *Ca*. Kinetoplastibacterium (D) Pea aphid, *Acyrthosiphon pisum*, and *Buchnera* endosymbiont shown within a bacteriocyte. (E) Representing both the red palm weevil, *Rhynchophorus ferrugineus*, and its *Nardonella* endosymbiont, and the cereal weevil, *Sitophilus*, and its endosymbiont *Sodalis pierantonius*. Shown within a bacteriocyte. (F) Mealybug, *Planococcus citri*, and its nested endosymbionts, *Ca*. Tremblaya princeps and *Ca*. Moranella endobia, shown within a bacteriocyte. (G) A legume and its rhizobia endosymbionts shown within a root nodule cell.

The ways in which host/symbiont coevolution transformed the envelope of more recently acquired endosymbionts from a defense surface into a symbiotic interface are barely understood. In many vertically transmitted endosymbionts, this transformation has been accompanied by the loss of genes involved in the generation of certain components of the bacterial envelope (Fig. 2), often accompanied by the gain of novel host‐derived membranes that became specialized for the symbiotic interaction (Fig. 3). In addition to the reduction of bacterial solute transporters (see above), some endosymbionts lost the ability to synthesize phospholipids (Fig. 2A,B), PG (Fig. 2A,C), or LPS (Fig. 2A,D); and loss of the Sec, Tat, and BAM complexes are not uncommon [78] (Fig. 2A,E). Most impressive is the pattern of gene loss within endosymbionts with extremely reduced genomes (< 0.2 Mbp) in which often all of the above‐mentioned envelope‐related functions are missing [78]. This indicates that these ‘endosymbionts’ completely depend on host‐derived lipids and presumably protein complexes to build their envelopes and equip them with functional proteins. How these losses are compensated by nuclear genes and the consequences of these losses for the characteristics of the endosymbiont envelope remain unclear, in particular concerning the permeability for proteins and metabolites. However, although a mechanistic understanding of protein import into bacterial endosymbionts is largely missing, there is experimental evidence that protein import occurs for a number of systems, which will be summarized in the next section.

## Protein targeting and import into bacterial endosymbionts

### Bacterial endosymbionts and evolving organelles in protists

#### Chromatophores of the cercozoan amoeba *Paulinella*

Cercozoan amoebae of the genus *Paulinella* contain two photosynthetic organelles, termed ‘chromatophores’ [79, 80], that evolved ~ 90–140 million years ago (MYA) from a cyanobacterium of the *Synechococcus/Prochlorococcus* clade [34, 81, 82]. Although chromatophores are ~ 140 times larger in volume than their free‐living relatives and fill up a large part of the host cell, they morphologically resemble cyanobacteria rather than plastids [83, 84]. Cell cycles of host and chromatophore are tightly synchronized and, during host cell division, one chromatophore segregates to the newly formed daughter cell [35] (Fig. 1A). Following chromatophore acquisition, the photosynthetic *Paulinella* lineage diversified into at least three species, *P. chromatophora*, *P. micropora*, and *P. longichromatophora* [80, 85, 86]. With a size of ~ 1 Mbp and containing 878–860 protein‐coding genes, chromatophore genomes lost around 2/3 of their original coding capacity [53, 80, 87, 88]. Today, the gene sets of the chromatophore and the host nucleus are highly complementary. Around 60 EGT and 170 HGT‐derived nuclear genes from bacteria other than the chromatophore ancestor contributed to this complementarity [45, 89, 90, 91, 92].

EGT‐derived genes include genes for the low molecular weight photosystem I subunits PsaE and PsaK (7.5 and 7.8 kDa, respectively). Isolation of photosystem I and radiolabeling studies in the presence of selective inhibitors of either prokaryotic or eukaryotic ribosomes demonstrated that the corresponding proteins are synthesized on eukaryotic ribosomes and imported into the chromatophore where they assemble with chromatophore‐encoded subunits into photosystem I complexes [93]. Immunogold transmission electron microscopy (TEM) using antibodies against PsaE revealed the protein not only in the chromatophore but also in the Golgi apparatus. This observation suggests that vesicular trafficking through the secretory pathway is involved in protein translocation across the outer of the two chromatophore envelope membranes, although PsaE and PsaK lack N‐terminal signal peptides (SPs) that typically target proteins into the secretory pathway. Based on electron micrographs, the outer chromatophore membrane has been interpreted as host‐derived whereas the cyanobacterial outer membrane has been lost [83, 94, 95] (Fig. 3A). This interpretation is in line with the loss of genes for LPS biosynthesis and export, BamA and other cyanobacterial outer membrane proteins from the chromatophore genome that apparently has not been accompanied by EGT [22, 53] (Fig. 2A).

**Fig. 4:**
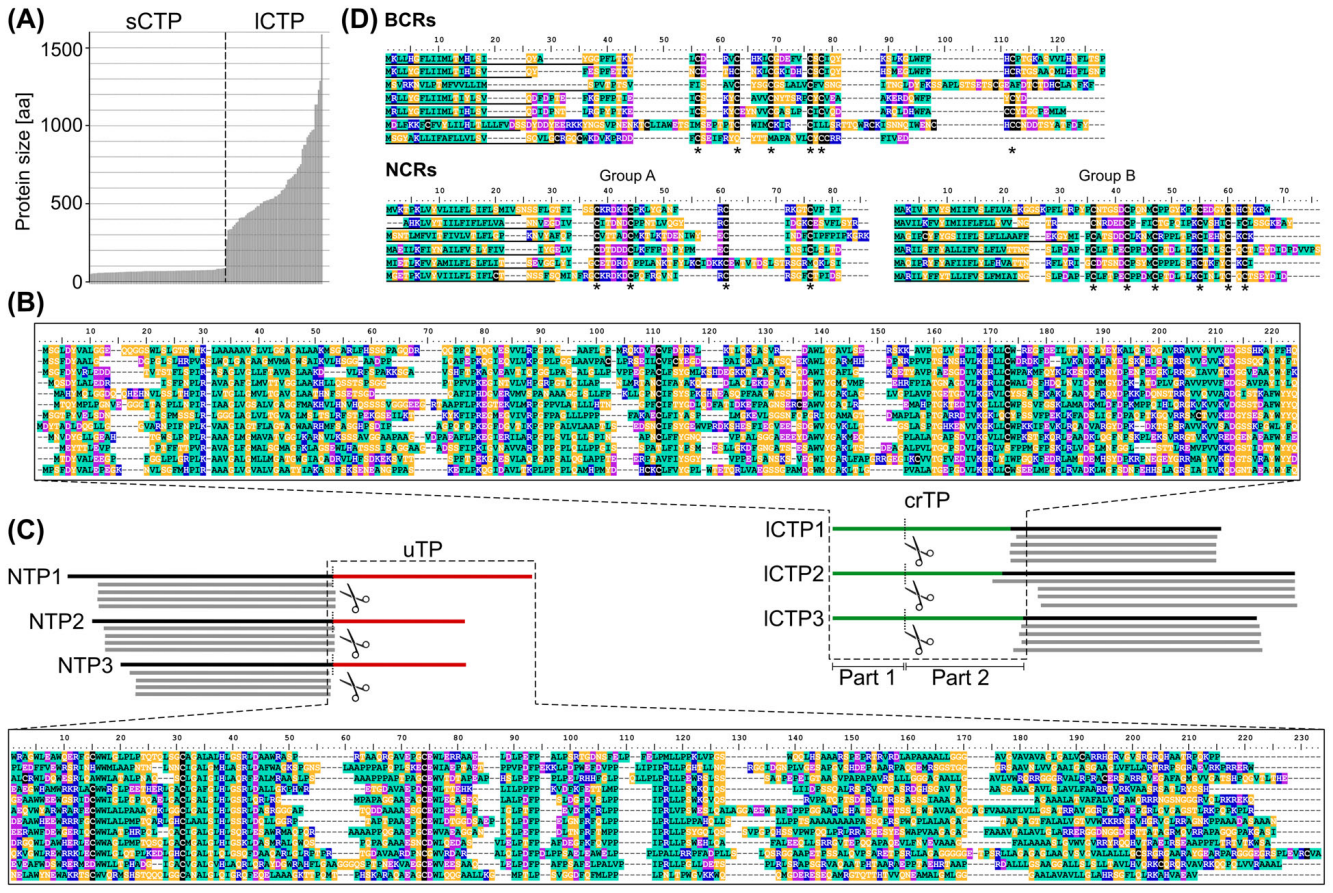
Comparison of endosymbiotic‐targeted proteins. (A) Chromatophore‐targeted proteins (CTP) in *Paulinella chromatophora* form two groups, short (< 90 aa) and long (> 250 aa) CTPs. (B) Schematic alignment of 3 lCTPs showing the characteristic bipartite N‐terminal extension (crTP, green). The zoom‐in shows a multiple sequence alignment of 12 representative crTPs. (C) Schematic alignment of 3 nitroplast‐targeted proteins (NTP) showing the characteristic C‐terminal extension (uTP, red). The zoom‐in shows a multiple alignment of 12 representative uTPs. Scissors symbols mark the targeting signal cleavage sites deduced by MS analyses in B and C. (D) Multiple sequence alignments of 7 BCRs from the aphid *Acyrthosiphon pisum* and NCRs from the legume *Medicago truncatula*. Six representative NCRs of group A (4 conserved cysteines) and group B (6 conserved cysteines) are shown. Signal peptides are underlined, and asterisks show conserved cysteine residues. For all alignments, amino acids are color‐coded in blue (positively charged; H, K, R), magenta (negatively charged; E, D), cyan (hydrophobic; A, P, W, I, L, M, V, F), orange (neutral; G, S, Y, N, Q, T), and black (cysteines; C). Sequence identifiers or accession numbers are, from top to bottom, for crTPs: scaffold7571‐size1527|m.60359; scaffold3807‐size2095|m.37686; scaffold10361‐size1249|m.74090; scaffold6875‐size1609|m.56608; scaffold8035‐size1476|m.62771; scaffold3865‐size2079|m.38081; scaffold5513‐size1797|m.48594; scaffold4638‐size1933|m.43151; scaffold2991‐size2309|m.31974; scaffold4337‐size1989|m.41170; scaffold2706‐size2392|m.29779; scaffold2155‐size2616|m.25388 [see ref. 21; available at PRIDE Repository (https://www.ebi.ac.uk/pride/archive/), accession number PXD006531]; for uTPs: KC1‐P2‐N_CL7753Contig1_1; KC1‐P2‐N_CL1062Contig1_1; KC1‐P2‐N_CL4024Contig1_1; KC1‐P2‐N_CL8449Contig1_1; KC1‐P2‐N_CL1190Contig1_1; KC1‐P2‐N_CL2249Contig1_1; KC1‐P2‐N_CL4289Contig1_1; KC1‐P2‐N_CL7819Contig1_1; KC1‐P2‐N_CL7868Contig1_1; KC1‐P2‐N_CL1296Contig1_1; KC1‐P2‐N_CL2661Contig1_1; KC1‐P2‐N_k25_Locus_10184_Trans [see ref. 23, available at Dryad, https://doi.org/10.5061/dryad.2z34tmptf]; for BCRs (AphidBase IDs): ACYPI32128; ACYPI38738; ACYPI44142; AK343177; AK339855; ACYPI49532; ACYPI45157; for NCRs (NCBI accession no.) group a: AFK48426.1; AES68835.1; ABS31393.1; RHN51124.1; AES98754.1; ABS31399.1 and group b: ABS31396.1; ABS31401.1; KEH26626.1; XP_039683422.1; KEH38199.1; AES78310.1. Sequence alignments were made with clustalx (B, C) or clustalw (D) and refined manually.

The global characterization of the chromatophore proteome by protein MS demonstrated that protein import is not restricted to a few low molecular weight proteins. Instead, hundreds of proteins that derive from EGTs and HGTs, but mostly from the host itself are imported and compensate for functions lost from the chromatophore genome or add novel functionality [21]. Interestingly, imported proteins form two classes (Fig. 4A). Whereas short chromatophore‐targeted proteins (sCTPs; < 90 aa) lack obvious targeting signals, long chromatophore‐targeted proteins (lCTPs; > 250 aa) carry a conserved N‐terminal ‘chromatophore transit peptide’ (crTP) (Fig. 4B). Some sCTPs share characteristics and form expanded families. Two of these families are characterized by specific cysteine motifs resembling the motifs found in nodule‐specific cysteine‐rich peptides (NCRs) of legumes and bacteriocyte‐specific cysteine‐rich peptides (BCRs) of aphids [22] (see below). Similar to NCRs and BCRs, at micromolar concentrations the purified proteins show antimicrobial activity against *Escherichia coli* and bind to bacterial membrane lipids *in vitro* [96]. Antimicrobial activity is not, however, restricted to cysteine motif‐containing sCTPs, but extends to sCTPs annotated as ‘4‐oxalocrotonate tautomerase’ and ‘DNA‐binding sCTPs’ (a protein family with > 200 members that bind to double‐stranded DNA) [96]. These annotations suggest functions in the chromatophore cytoplasm or at its nucleoid. Thus, it has been proposed that the lipid‐binding capacity and antimicrobial activity at (probably unphysiologically) high concentrations reflect a characteristic linked to the import mechanism of sCTPs rather than their physiological function [96].

Besides the expanded family of DNA‐binding sCTPs, also ~ 20% of lCTPs in both, *P. chromatophora* and *P. micropora*, have predicted functions associated with genetic information processing such as a DNA polymerase I (PolA), DNA helicases, a DNA ligase (LigA), predicted transcription factors, and ribosome‐associated proteins [21, 90]. Some of these proteins specifically fill gaps in chromatophore‐encoded processes. Additionally, within the lCTPs a family of ~ 20 octotrico peptide repeat proteins has been identified which serve in green algae as important nuclear factors regulating posttranscriptional steps of chloroplast gene expression [22]. Hence, it appears that in *Paulinella* the host gained control over DNA replication and can influence gene expression in the chromatophore.

The crTP targeting signal likely holds the key to understand the molecular mechanisms underlying the import of the long lCTPs into the chromatophore. MS characterization of the N‐termini of chromatophore‐targeted proteins demonstrated that the ~ 200 aa‐long crTP is bipartite [97]. In most lCTPs, only the first ~ 50 aa (crTP_part1_) are cleaved off during import (Fig. 4B). CrTP_part1_ contains signals that imply protein trafficking through the secretory pathway, suggesting that not only sCTPs but also lCTPs traffic via the Golgi into the chromatophore [93, 97]. Surprisingly, the remainder of the crTP (crTP_part2_) remains attached to the imported protein. Conserved secondary structure elements throughout crTP_part2_ suggest that it adopts a structural fold that possibly is responsible for translocating lCTPs across the PG layer and inner chromatophore membrane.

In sum, the chromatophore is a new genetically integrated photosynthetic organelle. The nucleus evolved considerable control over the chromatophore proteome not only by direct targeting of hundreds of nucleus‐encoded proteins into the chromatophore but additionally by controlling expression of chromatophore genes. Although many details on the protein import mechanism remain to be elucidated, it appears that it depends on the secretory pathway and that proteins exceeding a size cutoff of ~ 90 aa require a crTP_part2_ at their N terminus that provides a still unknown function. This import pathway, in which vesicular transport releases presumably folded proteins into the intermembrane space, is very different from TIC/TOC and TIM/TOM‐dependent protein import mechanisms into mitochondria and plastids. The translocation mechanism across the inner membrane and whether import clients are unfolded in this process is currently unclear.

#### Nitroplasts in the haptophyte alga *B. bigelowii*

Recently, evidence for the evolution of a second novel genetically integrated organelle has been found in the marine haptophyte *B. bigelowii* [23]. *B. bigelowii* and its relatives contain cyanobacteria of the UCYN‐A clade which coevolved with their host cells for ~ 91 million years and are related to a group of free‐living N_2_‐fixing cyanobacteria containing *Cyanothece*, *Gloeocapsa*, and *Crocosphaera* ssp. [98, 99]. Isotope tracer studies showed that UCYN‐A fixes N_2_ during the day and rapidly transfers it to the host, which in turn fixes CO_2_ into metabolites that are taken up by UCYN‐A [100, 101]. The symbiosis is widespread throughout global oceans and is an important contributor to marine N_2_‐fixation [100, 102, 103].

Within the host cytoplasm, UCYN‐A is enclosed in a host‐derived membrane in addition to its own two bacterial membranes and a PG layer [98, 104] (Fig. 3B). There is only a single UCYN‐A per *B. bigelowii* cell, which undergoes coordinated division with the host cell and is vertically transmitted [23, 104] (Fig. 1B). However, when nitrogen compounds are externally provided in culture, *B. bigelowii* can lose UCYN‐A [104]. The UCYN‐A genomes are ~ 1.4 Mbp in size and lost the genes for oxygenic photosynthesis and carbon fixation but retained a complete set of *nif* genes supporting N_2_ fixation [102, 105, 106].

Evidence for EGT has not been found in *B. bigelowii* [23, 104]. However, recently, protein MS of isolated UCYN‐A endosymbionts unveiled the import of > 350 nucleus‐encoded proteins, many of which compensate for functions lost from the cyanobacterial genome and complete metabolic pathways in UCYN‐A [23]. Some imported proteins apparently allow *B. bigelowii* to regulate specific aspects of genetic information processing in UCYN‐A. There is, for example, an imported methionine‐tRNA formyltransferase (Fmt) that is critical for initiating translation in prokaryotes as well as mitochondria and plastids and likely replaces the *fmt* gene which has been lost from the UCYN‐A genome [23]. 62% of the nucleus‐encoded proteins found enriched in the UCYN‐A fractions carry a conspicuous C‐terminal extension containing conserved amino acid motifs, that likely represents an import signal and thus has been termed the ‘UCYN‐A transit peptide’ (uTP) (Fig. 4C). By protein MS, no uTP‐derived peptides were identified suggesting that this presumptive targeting sequence is cleaved upon import.

Hence, the nitroplast shows a similar integration level as the chromatophore in *Paulinella*. Its ability to fix nitrogen makes it an exciting novel type of prokaryote‐derived eukaryotic organelle and demonstrates that organellogenesis events can be driven by the provisioning of functions other than aerobic respiration and photosynthesis. The uTPs point toward yet another mechanism for protein import into the novel organelle. The C‐terminal position of the uTP suggests that the proteins leave the ribosome fully folded before they are recognized as import clients, hence, although details are unknown, the underlying import mechanism is presumably very different from TIC/TOC and TIM/TOM‐dependent mechanisms.

#### Spheroid bodies in the diatom *Epithemia*

Rhopalodiacean diatoms house nitrogen‐fixing cyanobacterial endosymbionts that are designated ‘spheroid bodies’ or ‘diazoplasts’ and evolved from the same clade of free‐living cyanobacteria as *Braarudosphaera* nitroplasts [98, 107, 108]. Spheroid bodies are estimated to have been acquired 12–35 MYA [109, 110, 111]. Although they underwent significant genome reduction, with 3.0–2.5 Mbp, their genomes are (still) around twice the size as in UCYN‐A [112, 113, 114]. A considerable level of cellular integration is indicated by their vertical transmission during asexual as well as sexual reproduction [115, 116] and an apparently controlled number of spheroid bodies of between two and 16 (depending on the species and host cell size) [115]. However, in contrast to the nitroplast, a recent study found by protein MS only six host‐encoded proteins that were significantly enriched in spheroid bodies of the Rhopalodiacean *Epithemia clementina* [110]. Whether these proteins translocate across the bacterial membranes is unclear. However, since the host‐derived membrane surrounding the endosymbiont is lost during the isolation process, these proteins appear to be specifically targeted at least across this outermost host membrane. One of the endosymbiont‐targeted proteins (ETPs) is annotated as E3 ubiquitin ligase, the remaining proteins lack similarity to proteins of known function, and none of the ETPs are of bacterial origin. Therefore, their functions in the symbiotic interaction remain enigmatic.

Although there might be more ETPs that escaped detection (only 6.5% of the predicted nucleus‐encoded proteins were identified), spheroid bodies apparently have not reached the same level of genetic integration as chromatophores or nitroplasts (for which many more imported proteins were identified in MS studies that reached a similar coverage of the endosymbiont‐encoded proteins [21, 23]). As no genetic tools are available for *E. clementina* as of yet, determination of the subcellular localizations and cellular functions of the identified ETPs is challenging. Nevertheless, it would be of uttermost interest as it could help to unravel the molecular mechanisms that establish the tight integration of the spheroid body that apparently does not depend on massive protein import.

#### *Ca.* Kinetoplastibacterium spp. in symbiont‐harboring trypanosomatids

The Strigomonadinae are a subfamily of monoxenous trypanosomatids that live throughout their life cycle as commensals in the digestive tracts of insects. All members of this subfamily (comprising the genera *Angomonas*, *Strigomonas*, and *Kentomonas*) possess a vertically transmitted β‐proteobacterial endosymbiont, called *Ca*. Kinetoplastibacterium, that resides freely in the host cytoplasm and supplies the host with several metabolites and cofactors [33, 117, 118, 119]. Host/symbiont coevolution over ~ 40–120 million years resulted in genome reduction to a size of 0.8 Mbp [120, 121, 122]. An advanced level of cellular integration is indicated by a strict synchronization of the cell cycles of host and endosymbiont resulting in a single endosymbiont per host that divides just before the host cell divides [33, 37, 118] (Fig. 1C).

Protein MS of isolated endosymbionts in combination with *in vivo* localization studies of fluorescent fusion proteins, enabled by the development of genetic tools for this trypanosomatid, identified seven ETPs within *A. deanei* [123, 124]. Thus, also in *A. deanei*, genetic integration is not as advanced as for the chromatophore and UCYN‐A. The ETPs appear to be either host‐derived or represent orphan proteins of unclear provenance and most lack functional annotations. However, differential localizations of the ETPs within/around the endosymbiont suggest that they fulfill specific functions. ETP1 and ETP5 distribute over the endosymbiont envelope. ETP2, ETP7 (a predicted PG hydrolase), and ETP9 (a predicted dynamin‐like protein), localize at the endosymbiont division site. Since nucleus‐encoded PG hydrolases and dynamin‐like proteins are also required at the division site of mitochondria and/or plastids, it appears likely that these proteins provide *A. deanei* with nuclear control over endosymbiont division [125, 126, 127, 128]. In order to function as a PG hydrolase, ETP7 would have to translocate across the outer endosymbiont membrane to reach the PG‐containing periplasm. Finally, two ETPs, ETP3 and ETP8, showed a fluorescence pattern compatible with a localization in the symbiont cytosol plus localization over the Golgi. The outer membrane of *Ca*. Kinetoplastibacterium sp. is of bacterial origin (Figs 2A and 3C) but MS analyses detected phosphatidylcholine, a major phospholipid in eukaryotes that cannot be synthesized by endosymbiont‐encoded enzymes, as a major component of endosymbiont fractions [129] and TEM analyses captured vesicles that appear to fuse with the endosymbiont outer membrane of *Strigomonas culicis* [130]. These observations point toward a role of vesicular transport in host/symbiont communication, however, need to be corroborated by experimental data.

Additionally, *Ca*. K. crithidii encodes a single porin which likely contains 18 membrane‐spanning β‐strands and showed a slight preference for cations over anions in electrophysiological measurements [131]. Whether this porin serves only in nutrient exchange or enables protein translocation across the outer membrane is still to be elucidated.

At least one nuclear gene in *A. deanei* appears to result from EGT [46]. Its gene product, an ornithine cyclodeaminase that converts ornithine into proline, however, was not among the ETPs but acquired a new localization in the glycosomes, specialized peroxisomes in the trypanosomatids that tightly associate with the proline‐auxotroph endosymbionts [123].

Together, the results suggest that although massive protein import into the endosymbiont has not evolved in *A. deanei*, similar to the spheroid body, a number of host proteins have apparently started to interact with specific structures in the endosymbiont. The genetic tools available for this endosymbiotic system make *A. deanei* an ideal model to scrutinize the functions of these early ETPs and investigate how they provide the host with control over processes such as endosymbiont division (as suggested by the functional annotations and subcellular localizations observed for some of them).

### Nutritional endosymbionts in insects

There is a large diversity and prevalence of endosymbionts within insects [132]; they cover a wide range of functions, but have been particularly pivotal in enabling insects to survive on nutritionally imbalanced diets [133]. Many of these interactions are, therefore, essential and mutually obligate. Extreme genome reduction of the endosymbionts has occurred on multiple occasions [38, 39, 40]. In some cases, it has been shown that the host complements the endosymbiont directly with metabolic compounds, leading to mosaic pathways that cross multiple compartments [134]. In a few instances, however, there is evidence that proteins or polypeptides are transported into the endosymbiont, and these will be discussed below.

#### The pea aphid *Acyrthosiphon pisum*

The first example was found within the pea aphid *A. pisum*, which has harbored its γ‐proteobacterial endosymbiont, *Ca*. Buchnera aphidicola, for over 100 million years [135]. *Buchnera* is housed in specialized bacteriocyte cells, surrounded by two bacterial membranes, a PG layer, and one host symbiosomal membrane [136] (Figs 2A and 3D).

The *Buchnera* genome in *A. pisum* is 0.64 Mbp in size and biosynthetic pathways for many essential amino acids are split between host and symbiont [137]. Whereas protein MS analyses identified enzymes that were enriched in bacteriocytes and that mediate the biosynthesis of amino acids provided to the endosymbiont by the host, no host proteins appeared as reliably enriched in the endosymbionts themselves [138]. Additionally, expansions of amino acid transporter gene families have been described that are specifically expressed in the bacteriocytes [139]. These findings imply that pathways are linked on the metabolite level and large‐scale import of host‐encoded proteins into *Buchnera* does not occur. However, the total proteome analysis captured only 1940 out of 34 616 predicted aphid proteins leaving room for protein import that escaped detection.

Although no functional EGT occurred in the pea aphid, 12 nuclear genes apparently resulted from HGTs of diverse bacteria other than *Buchnera*, and seven of these have been found to be highly transcribed specifically in the bacteriocyte [47, 140]. The location of one of these proteins, the 21.2‐kDa protein RlpA4, was found to be restricted to the maternal bacteriocytes and specifically to the cytoplasm of the *Buchnera* cells within them, using immunoblot, immunofluorescence, and immunogold TEM analyses with antibodies raised against RlpA4 [141]. A 23 aa‐long SP likely directs the protein via the secretory pathway across the symbiosomal membrane [47, 140]. It remains unknown, however, how the protein proceeds to cross the bacterial envelope and what the precise function of RlpA4 is within the *Buchnera* cell [141].

Transcriptome comparison between bacteriocytes and entire aphids revealed among the 50 most highly over‐represented genes in bacteriocytes, 11 orphan genes encoding proteins with N‐terminal SPs. Seven of these genes encoded short peptides of 67–108 aa with six or eight cysteines that were termed ‘Bacteriocyte‐specific Cysteine‐Rich peptides’ (BCRs) (Fig. 4D), while the remaining secreted orphan proteins contain no cysteines and vary in length from 108 to 413 aa [142]. When externally applied to *E. coli* cells, four BCRs exhibited antimicrobial activity and were hypothesized to influence the endosymbiont in a similar manner to the NCR peptides in legumes (see below) [143]. The location of these peptides within the bacteriocytes and whether they are transported into the *Buchnera* cells have yet to be demonstrated. Nonetheless, it appears likely that, similar to RlpA4, some of these bacteriocyte‐specific secreted proteins translocate the bacterial envelope and serve as nuclear factors providing the host with control over certain biological processes of its endosymbiont.

#### The red palm weevil *Rhynchophorus ferrugineus*

Many weevils carry the γ‐proteobacterial endosymbiont *Ca*. Nardonella that resides free in the cytoplasm of the bacteriocytes of their larval bacteriome and is vertically transmitted from mother to offspring [144] (Fig. 3E). *Nardonella* coevolved with its weevil host for > 100 million years, which resulted in extreme genome reduction to 0.20–0.23 Mbp and 196–231 predicted protein‐coding genes in different weevil lineages [144, 145, 146, 147]. *Nardonella* lost genes for the biosynthesis of fatty acids, phospholipids, the BAM, Tat, and most of the genes for the Sec complex, only the PG biosynthetic enzymes are largely encoded [78] (Fig. 2A). Furthermore, while *Nardonella* endosymbionts encode minimal but supposedly complete gene sets for replication, transcription, and translation, they lost genes for almost all other metabolic pathways. Their genomes appear to be streamlined for a single biological function: provisioning tyrosine, an important precursor for cuticle formation in their weevil hosts [144]. However, a gene for the aminotransferase that catalyzes the last‐step in this pathway is missing in *Nardonella*. In the transcriptome of the black hard weevil *Pachyrhynchus infernalis*, two glutamate oxaloacetate transaminases, GOT1A and GOT2A, were identified that are preferentially expressed in bacteriocytes. Both proteins can convert the precursor 4‐hydroxy‐phenylpyruvate to tyrosine by transamination and were shown to be required for tyrosine production in the weevil by RNAi [144]. Recently, orthologs of these proteins, termed RfGOT1 and RfGOT2A, were identified in the red palm weevil *R. ferrugineus* where they are specifically upregulated in the bacteriome and required for tyrosine production as well [148]. Localization of both proteins in the *Nardonella* cytoplasm was observed by immunogold TEM and immunofluorescence experiments [148]. RfGOT1 and RfGOT2A have a size of 48.3 and 45.8 kDa, respectively, and carry no predicted SP at their N terminus, in line with the lack of a symbiosomal membrane. Further experiments are needed to confirm the import of these proteins; however, they suggest that other presumed‐bacteriocyte proteins should be tested for endosymbiont localization.

#### The cereal weevil *Sitophilus*

In the cereal weevil *Sitophilus*, the ancient *Nardonella* endosymbiont was replaced 20 MYA by another γ‐proteobacterial endosymbiont *Sodalis pierantonius*, also referred to as *Sitophilus* primary endosymbiont (SPE) [147, 149, 150]. SPE lies free in the cytoplasm of the bacteriocytes [151, 152] (Fig. 3E). Its genome of 4.51 Mbp is still relatively large, but shows signs of ongoing degeneration [150]. Within the *Sitophilus* bacteriocytes, an 8.6‐kDa antimicrobial peptide, ColA, was found to be highly expressed and has been implicated in the control of SPE [153]. Immunomicroscopy with antibodies against ColA showed the peptide localized within the endosymbiont cytoplasm in addition to specific host tissues [153]. Interestingly, the same antibodies also cross‐reacted with *Nardonella* isolated from the bacteriome of the palm weevil *R. ferrugineus*, suggesting that ColA has a broader impact on weevil symbioses. Far‐western blotting suggested that ColA gains access to the endosymbiont cytoplasm by interacting with Omp receptors, and RNAi revealed that ColA is involved in the regulation of endosymbiont growth and location [153]. *E. coli* cells challenged with ColA displayed cell giantism and polyploidy resembling the phenotype of the SPE in the symbiotic association. These results demonstrate that relatively recently captured endosymbionts can already be targeted by host effector proteins. Furthermore, they suggest that ColA is an ancient effector that has been used in weevils for symbiotic interactions long before the acquisition of SPE and that the intrinsic properties that enable ColA to interact with diverse bacteria make it a useful tool for gaining control over novel endosymbionts.

#### The mealybug *Planococcus citri*

The final case of protein import within insects was identified within the complex nested endosymbiosis of the mealybug *P. citri*. The bacteriocytes of the host house a β‐proteobacterium *Ca*. Tremblaya princeps, which itself houses a γ‐proteobacterium, *Ca*. Moranella endobia [28, 55]. The ancestor of *Tremblaya* was acquired ~ 100–200 MYA [154], whereas *Moranella* is one of several γ‐proteobacteria that have been independently acquired more recently in different *Tremblaya*‐harboring mealybug lineages [155]. In line with their different ages, *Tremblaya* contains an extremely reduced genome of 0.14 Mbp and the cells form 10–20 μm large spheres, which contain several *Moranella* cells with a genome size of 0.54 Mbp [28, 55]. *Tremblaya* is enclosed by three membranes, apparently two ‘bacterial membranes’ and a surrounding host vesicle, however, it has lost all genes required for the biogenesis of an envelope including the Sec, Tat, and BAM machineries (Figs 2A and 3F). Within *Tremblaya*, *Moranella* is surrounded by two bacterial membranes [28] (Fig. 3F). The overall patterns of gene loss and retention suggest that the two endosymbiont genomes complement one another to synthesize the essential amino acids the host requires [55]. Some of these mosaic pathways are additionally supplemented by host enzymes, the genes of which mostly originate from HGT events [42]. One such pathway is PG biosynthesis. A PG layer was found exclusively at the *Moranella*, but not the *Tremblaya*, cell envelope using nanoSIMS visualization of ^15^N D‐Ala [156]. Proteins involved in the biosynthesis of soluble PG precursors have been lost from the *Moranella* as well as *Tremblaya* genomes but are encoded as a result of HGTs in the *P. citri* mealybug genome [42]. These genes are, however, lineage specific and are not found in all nested endosymbiont‐hosting mealybugs. Using polyclonal antibodies and immunohistochemistry, one of these proteins, the D‐alanyl‐D‐alanine ligase MurF, was found to be located in the *Moranella* cytoplasm and, in a much lower concentration, to the bacteriocyte cytoplasm [156]. This finding suggests that PG precursors are synthesized by imported host‐encoded proteins within the *Moranella* cytoplasm, which itself encodes the last three steps of PG precursor biosynthesis, including the flippase MurJ that externalizes the membrane‐bound precursor lipid II (Fig. 2A,C). The mealybug MurF protein is a 56 kDa protein with a predicted 20 aa‐long SP, probably mediating translocation across the symbiosomal membrane. The transport mechanism by which MurF crosses the remaining four membranes separating the host and *Moranella* cytoplasm are as yet unknown as is the extent of protein import into *Moranella* and hence the level of genetic integration. However, the finding that a single nucleus‐encoded protein masters this spectacular targeting task translocating five membranes, makes it in our opinion likely, that a general targeting mechanism exists in *P. citri* that allows more nuclear proteins to reach *Moranella*.

### N_2_‐fixing rhizobia in legumes

Approximately 65 MYA members of the legume family established endosymbiotic associations with Rhizobia, bacteria, which induce the formation of root nodules in which they are enclosed in symbiosomal membranes and differentiate into N_2_‐fixing bacteroids [24, 157] (Fig. 3G). Rhizobia are a polyphyletic group of α‐ and a few β‐proteobacteria with genome sizes of ~ 5–10 Mbp [158]. Since legume seedlings are initially aposymbiotic, every plant generation has to establish the symbiosis anew from an environmental pool of bacteria. The microaerobic conditions inside the nodule protect the oxygen‐sensitive nitrogenase [159]. Bacteroid differentiation is accompanied by massive transcriptome changes including an induction of genes involved in N_2_‐fixation and a repression of many genes required for growth, including those for ribosomal proteins, and cell surface functions such as capsular polysaccharides, LPS, and outer membrane proteins [160, 161, 162].

In nodules of the inverted repeat‐lacking clade (IRLC) legumes, including *Medicago truncatula*, bacteroids undergo ‘terminal differentiation’ which includes cell elongation, polyploidization, and loss of reproductive capacity, whereas bacteroids in non‐IRLC legumes maintain their normal size, genome content, and reproductive capacity [163]. IRLC legumes, but not legumes without terminal bacteroid differentiation, encode short secreted peptides termed ‘nodule‐specific cysteine‐rich peptides’ (NCRs), which in *M. truncatula* form an expanded group of ~ 700 proteins. NCRs contain a conserved SP followed by highly divergent mature peptides of usually 35–55 aa length with four to six cysteine residues at conserved positions [164, 165, 166] (Fig. 4D). Their expression is almost exclusive to the nodules and is highly spatially resolved to functionally differentiated zones of the nodules [160, 161]. Within the nodule, NCRs are targeted via the secretory pathway to the bacteroids where they can interact with the membrane or different cytosolic components [167, 168, 169]. A unique ensemble of marker proteins distinguishes the symbiosomal membranes from other membrane‐bound compartments within the host and enables the targeted delivery of diverse plant‐derived proteins, marked with specific SPs, via the secretory pathway into the symbiosome [170, 171]. A nodule‐specific signal peptidase complex, containing the essential subunit DNF1, was identified in the model legume *M. truncatula*. In the *dnf1* mutant, symbiosome‐targeted proteins retain their SPs, are trapped in the ER, and rhizobia cannot differentiate into bacteroids [172].

The rhizobial transporter BacA has been implicated in NCR translocation across the bacteroid cytoplasmic membrane. BacA renders rhizobia sensitive to the peptide antibiotic bleomycin and is required for bacteroid differentiation in IRLC but not in non‐IRCL legumes [173]. BacA shares 64% sequence identity with the peptide transporter SbmA of *E. coli*. Determination of the structure of both proteins by cryo‐EM showed that they adopt the same overall fold with an outward‐open conformation and a large cavity that can accommodate diverse substrates. Furthermore, the structure suggested a proton‐driven mechanism for antimicrobial peptide import [174]. Since *bacA* mutants quickly lyse after endocytosis in NCR‐producing host plants, apparently, BacA facilitates not only the import of NCRs allowing them to reach their intracellular targets but concomitantly counteracts deadly amounts of membrane‐permeabilizing NCRs by defensive uptake, directing them away from the membrane to limit damage [175, 176].

The sheer number of different NCRs and lack of knowledge of their exact redox status *in planta* makes it challenging to dissect their exact mode of action in bacteroid differentiation, however, deletion of single NCRs can significantly impair bacteroid differentiation [166, 177, 178]. Described effects of NCRs on Rhizobia include inhibition of cell division, cell enlargement, genome endoreduplication, and manipulation of bacterial transcription and translation [168, 169, 179]. Furthermore, when externally added to cultured Rhizobia, NCRs can provoke features of terminal differentiation and some show antimicrobial properties, at minimal inhibitory concentration of ~ 2.5–12.5 μm, against diverse bacteria [169, 180].

Although rhizobia are controlled by a large arsenal of nucleus‐encoded proteins, we would not consider rhizobia as genetically integrated organelles. Their restriction to the root nodules and thus, exclusion from the plant reproductive organs, prevents vertical transmission of rhizobia. Hence, rhizobia retained the capacity to survive in the soil independently of their host plants and endosymbiont‐encoded functions have not been replaced by nuclear genes. Instead, rhizobia‐targeted legume proteins work as effectors that manipulate the rhizobial morphology and physiology and turn them into tiny N_2_‐fixing factories. This manipulation is an effective alternative to the genetic integration that is always linked to a permanent host/endosymbiont association with vertical transmission.

## Conclusions and future directions

Genomic characterization of countless bacterial endosymbionts led to an advanced understanding of the diversity of functions they provide to their hosts and the dynamics of their evolution. However, our insights into the cell biology of endosymbiotic associations, including a mechanistic understanding of host/symbiont interactions and the early steps in the transition from endosymbiont to organelle, is lagging far behind. This is at least partially due to the facts that endosymbiotic systems are extremely diverse, many cannot be cultivated easily, and for most, there are no, or no advanced, genetic tools available.

Only in two systems, namely *Paulinella* and *Braarudosphaera*, has the replacement of hundreds of endosymbiont proteins by nucleus‐encoded proteins, and hence a substantial level of genetic integration, been clearly established by the available data so far. However, it is certainly an oversimplification to assume that endosymbionts with extremely reduced genomes that lost essential genes for their central genetic processes or the capacity to build their own membranes can function by exchanging only metabolites with their host cells. The report of single compensatory host‐encoded proteins being imported into highly reduced bacterial endosymbionts in insects, where they replace lost functions, suggests that genetic integration of the endosymbiont has been achieved in additional systems.

However, to understand (a) how many highly reduced endosymbionts depend on the import of compensatory host‐encoded proteins, (b) the level of genetic integration of a given system, (c) how far metabolite exchange alone suffices for a stable endosymbiotic association, or (d) to which extent hosts depend additionally on the targeting of effector proteins into their endosymbionts to control their growth, physiology, and selective permeability, we need the global characterization of many more endosymbiont proteomes and functional studies on identified ETPs. Such studies from diverse systems representing different ages and levels of integration could help us to develop a universal model of the early steps in organellogenesis and understand how much room there is for variations on the theme. Furthermore, such data would help to identify common physicochemical features of the imported proteins (such as size, hydrophobicity, or conserved sequence elements) that in turn could help to identify components of the import pathways as well as constraints for import (such as size cutoffs dictated by the translocation systems in the different membranes or the PG layer itself).

A scenario for progressing organellogenesis that appears plausible in the light of the available, though limited, data is that a eukaryotic host cell starts relatively early on to target effector proteins into a bacterial endosymbiont, which can manipulate specific biological processes in the bacterium to stabilize the symbiotic association. Once a nucleus‐encoded protein with functional redundancy to the endosymbiont attains import capacity, deleterious mutations in the endosymbiont copy can get fixed and—if the resulting process cannot be replaced by metabolite import—the endosymbiont becomes dependent on the imported host protein. Processes that cannot be easily replaced by metabolite import would be, for example, genetic information processing from the endosymbiont genome, photosynthesis in a heterotrophic host with limited supply of organic nutrients, or nitrogen fixation in an oligotrophic environment. As soon as a general import mechanism with a specific targeting signal evolves, it likely spreads quickly and results in the import of hundreds of proteins into the new organelle and hence, increases over time the degree of genetic integration. It is important to note that EGT is not a precondition for the genetic integration of an endosymbiont. In *Paulinella*, EGT played only a circumstantial role in genetic integration of the chromatophore, whereas in *Braarudosphaera* no EGT at all was required. The likelihood that EGT contributes to the set of endosymbiont‐targeted support proteins depends on (a) the number of endosymbiont genes that are being functionally replaced by imported proteins, (b) the access of the host to alternative sources of genetic material via HGT, and (c) the nuclear integration rate of endosymbiont‐derived chunks of DNA of a sufficient size, which depends on diverse factors intrinsic to the biology of the host and the endosymbiont.

Although the formal possibility exists that mRNA import, not protein import, establishes the endosymbiont's dependence on nuclear genes, the observed conservation of different targeting sequences (SPs, crTPs, uTPs) on the amino acid level, plus experimental evidence in some systems (such as legumes and *Paulinella*, see above), renders this scenario very unlikely. However, dissecting the molecular mechanisms underpinning the protein translocation across the endosymbiont‐surrounding membranes remains very challenging. A key advancement will be the detailed characterization of the proteome and protein complexes in these membranes and the interactions between some of their components and imported proteins. Leverage would be provided by the further development of model systems and genetic tools for these systems. Thus, it will take time and effort before a mechanistic understanding of the diverse mechanisms of protein import that are utilized in different endosymbiotic associations becomes available. However, research in this direction holds the promise to unravel a plethora of novel biological mechanisms for targeting, insertion, and translocation of proteins with different characteristics and folding states to, in, and across biological membranes. Moreover, this will provide vital insights into the circumstances promoting organellogenesis events. It is an exciting time in the field of endosymbiosis research, and the more systems we identify on the endosymbiont to organelle spectrum and characterize in depth, the deeper we can expand our understanding of this complex evolutionary transition.

## Conflict of interest

The authors declare no conflict of interest.

## Author contributions

MESS, MLS, LK, and ECMN wrote the manuscript.
